# Cell Plasticity and Genomic Structure of a Novel Filterable *Rhizobiales* Bacterium that Belongs to a Widely Distributed Lineage

**DOI:** 10.3390/microorganisms8091373

**Published:** 2020-09-07

**Authors:** Ryosuke Nakai, Takeshi Naganuma, Nozomi Tazato, Sho Morohoshi, Tomomi Koide

**Affiliations:** 1 Microbial Ecology and Technology Research Group, Bioproduction Research Institute, National Institute of Advanced Industrial Science and Technology (AIST), 2-17-2-1 Tsukisamu-higashi, Toyohira-ku, Sapporo 062-8517, Japan; 2 Graduate School of Integrated Sciences for Life, Hiroshima University, 1-4-4 Kagamiyama, Higashihiroshima 739-8528, Japan; takn@hiroshima-u.ac.jp; 3 Technical Department, TechnoSuruga Laboratory Co. Ltd., 330 Nagasaki, Shimizu-ku, Shizuoka 424-0065, Japan; nt_1002@tecsrg.co.jp (N.T.); sm_1050@tecsrg.co.jp (S.M.); tk_1046@tecsrg.co.jp (T.K.)

**Keywords:** bacteria, filterable bacteria, phylogeny, novel lineage, genome, nitrogen cycling

## Abstract

*Rhizobiales* bacterium strain IZ6 is a novel filterable bacterium that was isolated from a suspension filtrate (<0.22 µm) of soil collected in Shimane Prefecture, western Japan. Additional closely related isolates were recovered from filterable fractions of terrestrial environmental samples collected from other places in Japan; the Gobi Desert, north-central China; and Svalbard, Arctic Norway. These findings indicate a wide distribution of this lineage. This study reports the cell variation and genomic structure of IZ6. When cultured at lower temperatures (4 °C and 15 °C), this strain contained ultra-small cells and cell-like particles in the filtrate. PacBio sequencing revealed that this chromosome (3,114,641 bp) contained 3150 protein-coding, 51 tRNA, and three rRNA genes. IZ6 showed low 16S rRNA gene sequence identity (<97%) and low average nucleotide identity (<76%) with its closest known relative, *Flaviflagellibacter deserti*. Unlike the methylotrophic bacteria and nitrogen-fixing bacteria in related genera, there were no genes that encoded enzymes for one-carbon-compound utilization and nitrogen fixation in the IZ6 genome; the genes related to nitrate and nitrite reductase are retained and those related to the cell membrane function tend to be slightly enriched in the genome. This genomic information helps elucidate the eco-physiological function of a phenotypically heterogeneous and diverse *Rhizobiales* group.

## 1. Introduction

Filter sterilization using a small pore size filter (approximately 0.2 µm) is commonly used in various scientific fields, and medical and industrial processes. However, previous reports demonstrated that a wide range of bacteria can pass through such micropore filters (hereafter, filterable bacteria) and exist in various environments, including seawater [1,2], lake water [3,4], deep-sea hydrothermal fluids [5,6], and groundwater [7,8]. Efforts to cultivate filterable bacteria have yielded previously uncultured members, such as the cosmopolitan freshwater *Actinobacteria* [9] and candidate phylum terminate group 1 (TG1; described as *Elusimicrobia* [10]). Filtration is considered an effective approach for removing readily cultivable bacteria that overgrow slow-growing bacteria [11]. The filterable bacteria cultivated to date include ultra-small bacteria (e.g., ultramicrobacteria, which are defined as having a cell volume of <0.1 μm^3^ [12]), miniaturized ultramicrocells induced by external factors, and slender filamentous bacteria with small cell widths [13].

During the course of mining filterable bacteria, we isolated a novel *Rhizobiales* bacterium, strain IZ6, from a suspension filtrate of soils collected in Shimane Prefecture, western Japan [14]. Intriguingly, this strain was distantly related to members of known genera with low 16S rRNA gene sequence similarities (<97%), but was closely related to other strains isolated from the filtered fractions of various environmental samples, including bark, sand, soil, and travertine, which were collected from other places in Japan; the Gobi Desert, north-central China; and Svalbard, Arctic Norway [14]. These results indicate this unique lineage shows a wide distribution in terrestrial habitats. The cell size is relatively large compared with known ultramicrobacteria; this strain may possess ultramicrocells or may be a slender bacterium. However, cell size variation and eco-physiological potential could not fully be characterized. Here, we compensated for the limited knowledge by analyzing the detailed cell morphology and genomic traits of this widely distributed filterable strain.

## 2. Materials and Methods

### 2.1. Test for Cell Morphological Change and Filterability

IZ6 grows best when cultivated in R2A Broth “DAIGO” (Nihon Pharmaceutical Co., Ltd., Tokyo, Japan) at 25 °C. This study examined temperature-induced morphological changes. Cell morphology of IZ6 was observed by microscopy (Olympus BX-50F4, Olympus Optical Co. Ltd., Tokyo, Japan) with a Gram-staining kit (Favor G “Nissui”; Nissui Pharmaceutical Co., Ltd., Tokyo, Japan) after cultivation at 25 °C for 2 weeks, 25 °C for 2 weeks followed by 4 °C for 3 weeks, and 15 °C for 3 weeks. Culture fluid was filtered through a 0.22-μm filter (Millex-GV Syringe Filter Unit SLGV033RS; Merck Millipore, Tokyo, Japan), and the cells in the filtrate were also observed. Cell volume was calculated with the formula described in [15].

### 2.2. Genomic Sequencing, Assembly, and Annotation

IZ6 was cultivated in R2A Broth “DAIGO” at 25 °C. After 2–3 weeks of incubation, the OD_600_ value increased to 0.08–0.09 and the cultured cells were used for DNA extraction. DNA extraction from cell pellets was performed using a QIAGEN Genomic-Tip 500/G column (Qiagen, Tokyo, Japan) and Genomic DNA Buffer Set (Qiagen) according to manufacturer’s instructions. DNA purification was conducted with AMpure XP (Beckman Coulter, Tokyo, Japan). DNA fragmentation was performed with a g-TUBE (Covaris Inc., Woburn, MA, USA). The DNA library for PacBio sequencing was prepared with the SMRTbell Template Prep Kit 1.0 (Pacific Biosciences, MenloPark, CA, USA) and DNA/Polymerase Binding Kit P6 v. 2 (Pacific Biosciences). Whole-genome sequencing was performed on a PacBio RS II sequencer (Pacific Biosciences) using DNA Sequencing Reagent 4.0 v. 2 (Pacific Biosciences). De novo assembly was performed with RS_HGAP_Assembly.3. Possible genomic contamination was checked by ContEst16S [16]. The genome was annotated by the whole genome analysis pipeline in EzBioCloud [17]. Briefly, protein-coding sequences (CDSs) were predicted by Prodigal 2.6.2 [18]. tRNA was searched using tRNAscan-SE 1.3.1 [19], and rRNA and other non-coding RNAs were searched by covariance model search in the Rfam 12.0 database [20]. CRISPRs were detected using PILER-CR 1.06 [21] and CRT 1.2 [22]. The CDSs identified were classified into clusters of orthologous groups (COGs) based on their roles according to the reference database (EggNOG 4.5 [23]) and compared with Swiss-Prot [24], KEGG [25], and SEED databases [26] using UBLAST [27]. After further functional annotation by DFAST [28] of DDBJ, the IZ6 genome sequence was deposited in the DDBJ/ENA/GenBank database under accession no. AP023361. The BioProject and BioSample numbers are PRJDB10335 and SAMD00238716, respectively.

### 2.3. Phylogenetic and Comparative Genomic Analyses

A phylogenetic tree of near-full-length 16S rRNA gene sequences was constructed by MEGA X [29] with the neighbor-joining method [30], 1000 bootstrap replicates [31], and the Kimura 2-parameter model [32]. For comparative genomic analysis, sequences of four related strains that were selected based on the 16S rRNA gene-based phylogenetic tree (*Flaviflagellibacter deserti* CPCC 101076, *Hansschlegelia zhihuaiae* S113^T^, *Methylosinus trichosporium* OB3b^T^, and *Pleomorphomonas diazotrophica* R5-392^T^), were obtained from the EzBioCloud database [17]. The genome-wide average nucleotide identity (ANI) was calculated by OrthoANI [33]. Pan-genome orthologous groups (POGs) were identified by a combined reciprocal best-hit method using UBLAST [34] with an e-value threshold of 1 × 10^−6^ and an open reading frame-independent method using nucleotide sequences [35] with a cut-off of at least 70% sequence coverage. After grouping, partial short gene sequences were targeted and used for clustering analysis against the identified POGs using UCLUST [27] with a cut-off of ≥95% sequence identity. The heatmap and unweighted pair group method with arithmetic mean (UPGMA) dendrogram were constructed with the calculated POGs. The Venn diagram of shared and specific CDSs was visualized by jvenn [36].

## 3. Results and Discussion

### 3.1. Temperature-Induced Cell Morphological Change

The existence of filterable cells in IZ6 varied with culture temperature (Table 1; note that micrographs of the stained cells are shown in Figure S1). This strain is a filterable bacterium; however, when cultured at 25 °C, the cells before filtration were larger than the micropore filter pore size (approximately 0.2 µm), and no cells were observed in the filtrate. The cell volume (~0.36 µm^3^) was greater than that (<0.1 µm^3^ [12]) defined for ultramicrobacteria. However, ultramicrobacteria-like cells were observed in the filtrate when further incubated at 4 °C. Such small cells were also found in the filtrate when initially cultured at 15 °C. These small cells may have included dead cells and/or cell fragments; however, a previous study confirmed regrowth of IZ6 cells that remained in the filtered fraction [14]. Therefore, it is logical to assume that viable small cells remained; future studies should confirm how small cells in the filterable population regrow. Moreover, close relatives of IZ6 were isolated from the filtrates of terrestrial samples collected worldwide [14]; the cell plasticity of members in this lineage may allow them to “squeeze” through the micropore filters.

External factors are well known to affect bacterial cell size. Other studies clearly showed that starvation and simulated natural environments induced cell miniaturization (~50% reduction in size) of *Staphylococcus aureus* [37] and *Pseudomonas syringae* pv. *syringae* [38]. However, the temperatures examined in this study were reported to affect the growth rate without cell size reduction in some bacteria (e.g., *Salmonella typhimurium* [39]). The IZ6 cells before filtration also did not significantly differ between cultures at 25 °C and 15 °C, but they contained filterable cells at lower temperatures. Although the mechanism by which such small cells occur is unclear, ultra-small cells may be the starvation form [40] or have the advantage of a larger surface-to-volume ratio, which allows more efficient uptake of surrounding nutrients [12]. Note that because IZ6 growth was slow even in the best growing medium, it was difficult to verify morphological change under different nutrient concentrations (data not shown). Although further study is required to address such morphological changes, IZ6 was found to possess filterable ultra-small cells induced by culture temperature.

### 3.2. Genome Structure and Phylogenetic Relationships

**Figure 1:**
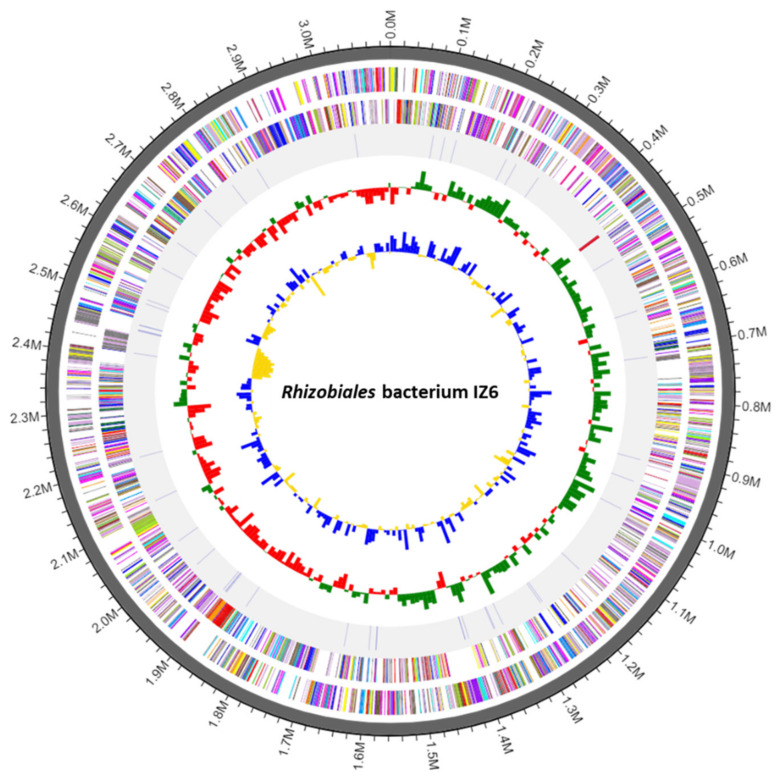
Genomic map of the *Rhizobiales* bacterium IZ6 chromosome. From outside to center: genes, specifically protein-coding sequences, on the forward strand are colored by clusters of orthologous group (COG) functional categories; genes on the reverse strand; rRNA and tRNA; guanine−cytosine (GC) skew metric (mean GC-skew value was used as a baseline, with higher-than-average values in green and lower-than-average values in red); and GC ratio metric (mean GC ratio was used as a baseline, with higher-than-average values in blue and lower-than-average values in yellow).

We determined the complete genome sequence of *Rhizobiales* strain IZ6. The genome consisted of a 3,114,641-bp (approximately 3.1-Mb) chromosome with 158-fold genome coverage. No genome contamination was confirmed by ContEst16S. The guanine+cytosine (G+C) content was 62.2 mol%. The chromosome contained 3150 CDSs, 51 tRNA genes, and three rRNA genes. The coding ratio was 91.9%. No CRISPRs were identified in the genome. All identified CDSs are listed in Table S1. The detailed genomic map with COG function categories is displayed in Figure 1 (COG sub-categories and their color codes and gene information are shown in Figure S2).

**Figure 2:**
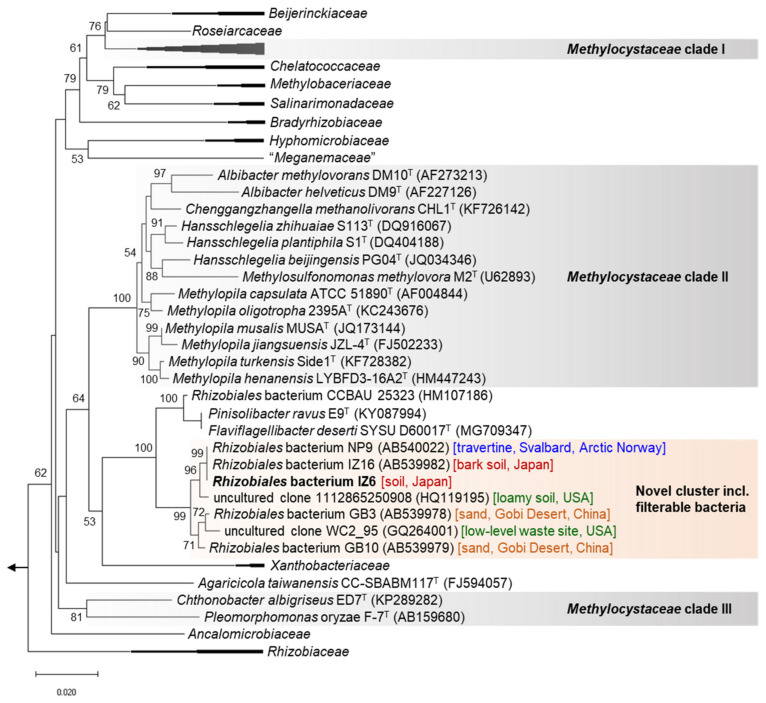
Phylogenetic tree based on near full-length 16S RNA gene sequences of IZ6 and its close relatives in the order *Rhizobiales*. The evolutionary relationship was inferred using the neighbor-joining method with the Kimura 2-parameter model. There was a total of 1128 positions in the final dataset. Members of the *Terasakiella* genus (*Methylocystaceae* Clade IV) were used as an outgroup. Five genera (*Bartonella*, *Brucella*, *Phyllobacterium*, *Mabikibacter*, and *Notoacmeibacter*) that are relatively distantly related to IZ6 are not described here. Accession numbers of nucleotide sequences registered in the DDBJ/ENA/NCBI databases are shown in parentheses. For the novel cluster presented in this study, the isolation source and country are shown in square brackets. Bootstrap values >50% based on 1000 replicates are shown at the nodes. Scale bar, 0.020 nucleotide substitutions per site.

The full-length 16S rRNA gene sequence (1480 bp) of IZ6, which was retrieved from the genome, showed low sequence identity (96.9%) with the nearest type strain, *Flaviflagellibacter deserti* SYSU D60017^T^, in the order *Rhizobiales* (class *Alphaproteobacteria*), as recently described [41]. Figure 2 shows the phylogenetic placement of IZ6 based on the 16S rRNA gene-based phylogenetic tree. Consistent with our previous research [14], IZ6 formed an independent cluster that was separate from *F. deserti* and its relatives, which included other filterable strains that were isolated from Japan; the Gobi Desert, north-central China; Svalbard, Arctic Norway; and an uncultured environmental clone (DDBJ/ENA/NCBI accession no. GQ264001 [42]) from simulated a low-level-radioactive-waste site in the USA. Moreover, this study showed that an additional uncultured loamy soil clone (HQ119195 [43]) belonged to this independent cluster. The phylogenetic position indicated that IZ6 is a phylogenetically novel bacterium.

*Flaviflagellibacter deserti* SYSU D60017^T^, the closest strain of IZ6, was first described as a novel genus and species related to *Methylocystaceae* clade II but with low sequence identity (<94%) to known genera [41]. This type strain was not isolated from the filtered fraction of the environmental sample. The *Methylocystaceae* lineage contains four clades with different positions on the phylogenetic tree [41,44]: clade I (type II methanotrophs, including the genera *Methylocystis* and *Methylosinus*), clade II (methylotrophs, including the genera *Albibacter*, *Chenggangzhangella*, *Hansschlegelia*, *Methylosulfonomonas*, and *Methylopila*), clade III (non-methanotrophs, including the genera *Chthonobacter* and *Pleomorphomonas*), and clade IV (non-methanotrophs, including the genus *Terasakiella*) (Figure 2). Note that clade IV is relatively distant from the other clades. These clades may need to be reclassified and reorganized in future taxonomic studies. Moreover, as discussed below, the IZ6 genome lacks gene sequences (e.g., methanol dehydrogenase) related to the utilization of one-carbon compounds, which are characteristic to methylotrophs. This clearly indicates that IZ6 is a non-methylotrophic bacterium, unlike clade II members.

### 3.3. Ecophysiological Characteristics and Potential Inferred from Comparative Genomics

**Figure 3:**
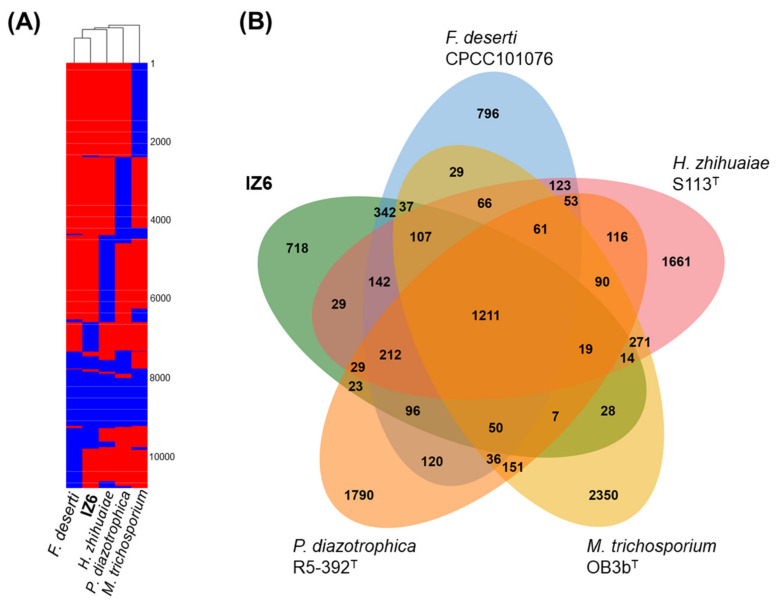
(**A**) Heat map and unweighted pair group method with arithmetic mean (UPGMA) dendrogram based on gene content (presence/absence) of all protein-coding sequences (CDSs) of strain IZ6 and four related strains (*Flaviflagellibacter deserti* CPCC 101076, *Hansschlegelia zhihuaiae* S113^T^, *Methylosinus trichosporium* OB3b^T^, and *Pleomorphomonas diazotrophica* R5-392^T^) in order *Rhizobiales* (class *Alphaproteobacteria*). The presence and absence of genes (pan-genome orthologous groups) on the heatmap are indicated in blue and red, respectively. (**B**) Venn diagram of shared and specific CDSs among the five strains.

Comparative genomic analysis between IZ6 and four related *Rhizobiales* bacteria was conducted to illustrate the phylogenomic relationship and potential ecological function of IZ6. IZ6′s ANI was low (<76%), even with *F. deserti*, its closest relative based on 16S rRNA gene sequences. Considering the threshold for taxonomic assignment [45], the genome-wide low ANI also supported the taxonomic novelty of IZ6. Note that the genome size (3.1 Mb) of strain IZ6 tended to be smaller than that of other *Rhizobiales* members (average 5.3 Mb; Figure S3), with smaller genomes mainly belonging to pathogenic groups (e.g., *Bartonella*, *Brucella*, and “*Candidatus Liberibacter*”). The heatmap dendrogram based on gene content (presence/absence) of all CDSs was visualized in Figure 3A. Consistent with the phylogenetic and phylogenomic relationships, IZ6 was closely aligned with *F. deserti* on the dendrogram.

The *Rhizobiales* group is phenotypically highly heterogeneous and contains methanotrophs, methylotrophs, and nitrogen fixers [41,44]. Based on the POGs among the compared strains, the IZ6 genome lacked gene sequences related to methanol dehydrogenase and methane monooxygenase for one-carbon compound metabolism. Moreover, unlike nitrogen fixers of related genera (e.g., *Rhizobium* and *Pleomorphomonas*), the genome lacked nitrogenase for nitrogen fixation to convert atmospheric nitrogen gas to ammonia. These genomic features are shared with *F. deserti* [41]. Alternatively, as with the other selected strains, three key genes (nitrate reductase/*nasA* and nitrite reductase/*nirA*/*nirB*) for assimilatory nitrogen reduction to ammonium were detected in the IZ6 genome. Our comparative genomic analysis also identified 718 IZ6-specific CDSs (Figure 3B). The specific CDSs contained genes that encode some decarboxylases (e.g., oxalyl-CoA decarboxylase), cell membrane-related proteins and transporters, and multiple hypothetical proteins (note that the all specific CDSs are listed in Table S2). In this context, the COG subcategory M (Cell wall/membrane/envelope biogenesis) in the IZ6 genome accounted for 5.4% of the total and tended to be higher than those (3.9–4.9%) in other genomes, with the exception of 5.7% in *M. trichosporium* genome (Table S3). Furthermore, the proportion of genes not matched to existing COG subcategories was the highest (12.8%) among the genomes compared (Table S3).

Based on the genes detected, the overlooked filterable agents, including IZ6, are at least expected to be involved in the nitrogen cycle in their original habitats. Because the IZ6 genomic data is an unexplored part of the diverse *Rhizobiales* members, further genome-wide and transcriptomic analyses will elucidate their phenotypic diversity and ecological functions.

## Figures and Tables

**Table 1:** Cell size before and after 0.22-µm filtration under different culture temperatures.

Temperature	Cell Length × Width and Volume	
	Before Filtration	After Filtration ^1^
25 °C for 2 weeks	1.5–2.0 × 0.4–0.5 µm	no cell
	0.17–0.36 µm^3^	
25 °C for 2 weeks followed by 4 °C for 3 weeks	2.0–2.5 × 0.4–0.5 µm	1.0 × 0.2–0.3 µm
	0.23–0.46 µm^3^	0.03–0.06 µm^3^
15 °C for 3 weeks	1.5–2.0 × 0.3–0.5 µm	0.5–1.0 × 0.2–0.3 µm
	0.10–0.36 µm^3^	0.01–0.06 µm^3^

^1^ Cells in the filtrate may have contained dead cells and/or cell fragments.

## Supplementary Materials

The following are available online at https://www.mdpi.com/2076-2607/8/9/1373/s1, Figure S1: Micrographs of Gram-stained cells of strain IZ6 before and after 0.22 µm filtration. The cells were cultured under different temperature conditions: 25 °C for 2 weeks (panel A), 25 °C for 2 weeks followed by 4 °C for 3 weeks (panels B and C), and 15 °C for 3 weeks (panels D and E). Figure S2: Clusters of orthologous group (COG) sub-categories and category distribution of the filterable *Rhizobiales* bacterium IZ6 genome. COG color codes and their gene information are shown in the right panel. The proportion of genes not matched to COG subcategories was 12.8% (see Table S3 for details). Figure S3: Genome size and guanine+cytosine (G+C) content of all genomes of *Rhizobiales* members currently deposited in the Integrated Microbial Genomes and Microbiomes (IMG/M) database. The genomes of 2619 *Rhizobiales* members registered in IMG/M [46] as of August 22, 2020, were used in this analysis. Values of strain IZ6 are indicated in a yellow-filled circle. Only genomes larger than 1 Mb are shown. Table S1: All protein-coding sequences (CDSs) identified in the *Rhizobiales* strain IZ6 genome. Table S2: All specific protein-coding sequences (CDSs) detected in the *Rhizobiales* strain IZ6 genome. The genome data were compared with those of four related strains (*Flaviflagellibacter deserti* CPCC 101076, *Hansschlegelia zhihuaiae* S113^T^, *Methylosinus trichosporium* OB3b^T^, and *Pleomorphomonas diazotrophica* R5-392^T^). Table S3: Number of genes per clusters of orthologous group (COG) subcategory in the *Rhizobiales* strain IZ6 genome. The COG data were compared with those of four related strains (*Flaviflagellibacter deserti* CPCC101076, *Hansschlegelia zhihuaiae* S113^T^, *Methylosinus trichosporium* OB3b^T^, and *Pleomorphomonas diazotrophica* R5-392^T^).
