# Supplementary figures and images for: Cell Plasticity and Genomic Structure of a Novel Filterable Rhizobiales Bacterium that Belongs to a Widely Distributed Lineage
Source: Microorganisms. 2020 Sep 7;8(9):1373. doi: 10.3390/microorganisms8091373 (PMC7564735; doi:10.3390/microorganisms8091373)

## Before filtration

## After filtration

25°C for 2 weeks

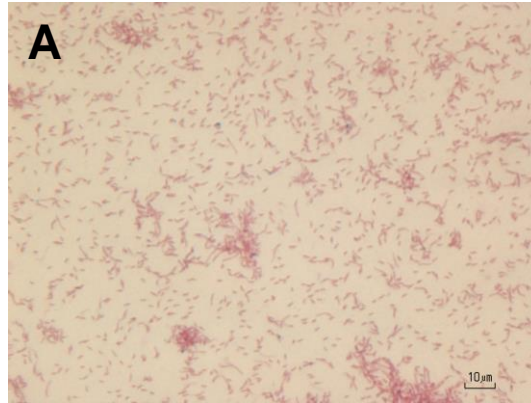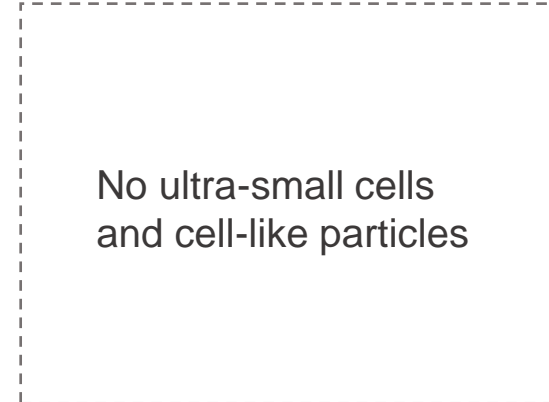

25°C for 2 weeks  
followed by  
4°C for 3 weeks

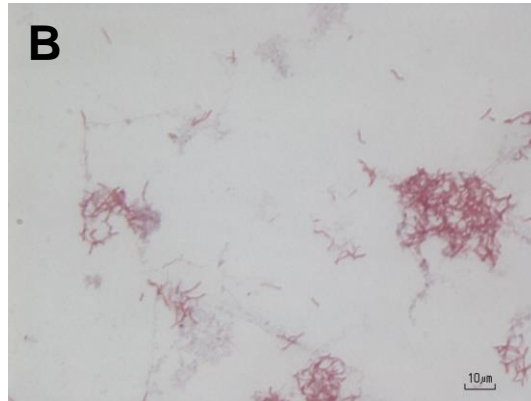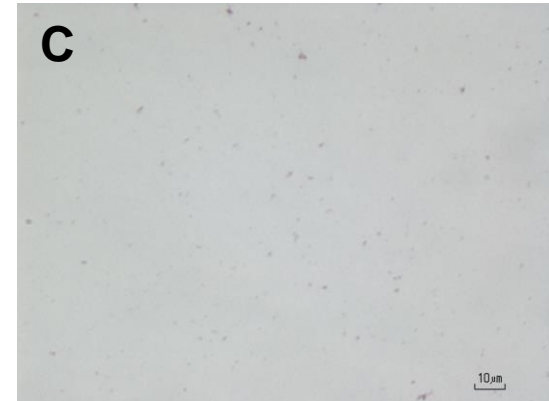

15°C for 3 weeks

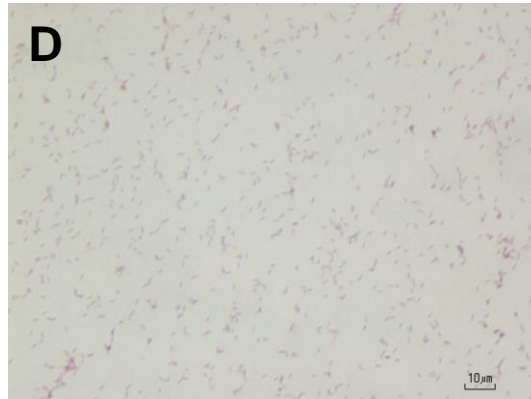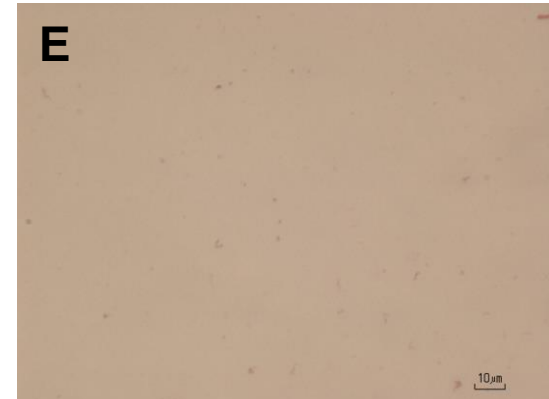

Supplement: Supplementary file 1 [file microorganisms-08-01373-s001.zip › SupplementaryFiles.v2/2020-08-22_Microorganisms.IZ6.Supplement.Fig1.pdf]

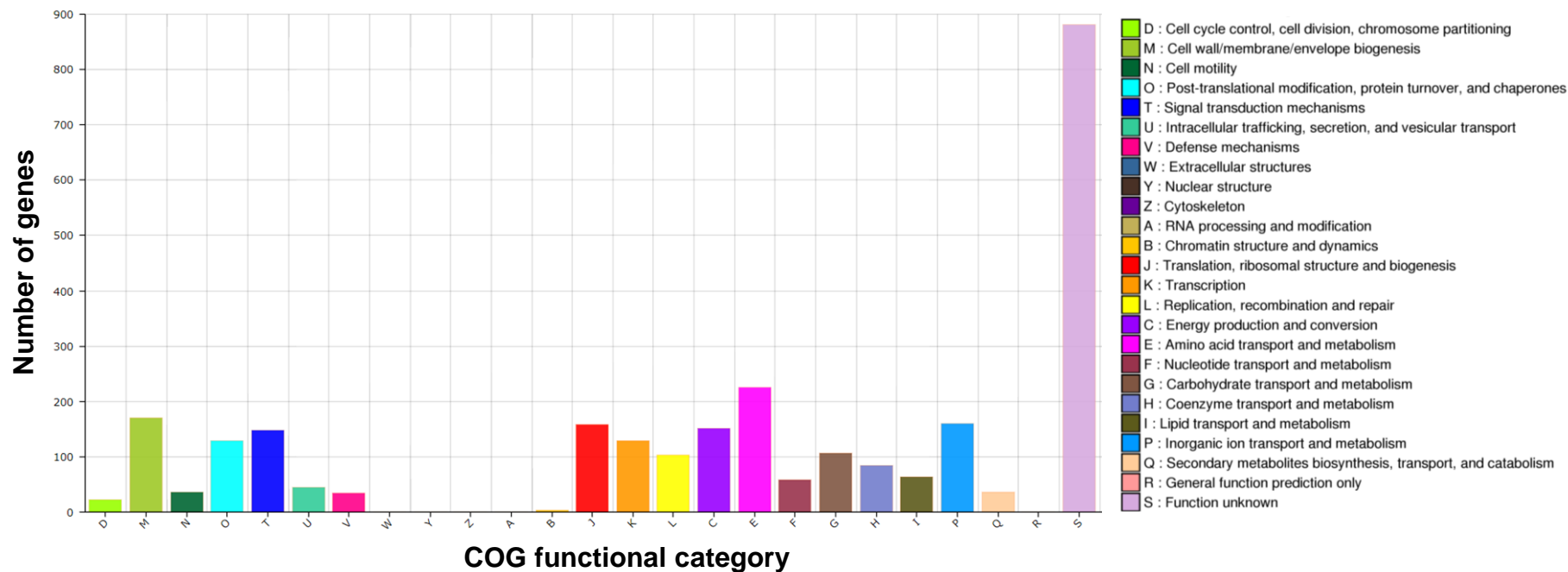

Supplement: Supplementary file 1 [file microorganisms-08-01373-s001.zip › SupplementaryFiles.v2/2020-08-22_Microorganisms.IZ6.Supplement.Fig2.pdf]

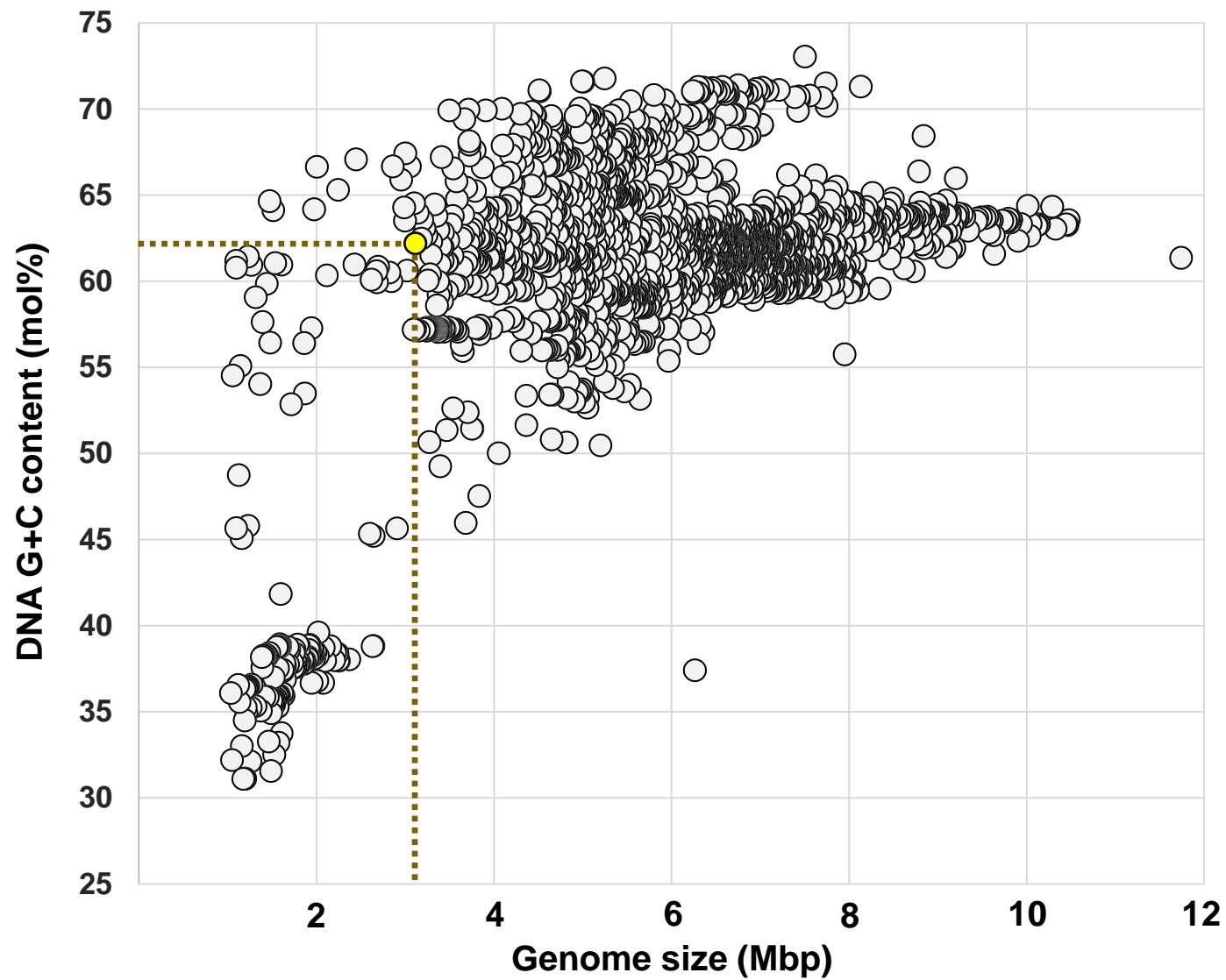

Supplement: Supplementary file 1 [file microorganisms-08-01373-s001.zip › SupplementaryFiles.v2/2020-08-23_Microorganisms.IZ6.Supplement.Fig3.pdf]
